# NeVOmics: An Enrichment Tool for Gene Ontology and Functional Network Analysis and Visualization of Data from OMICs Technologies

**DOI:** 10.3390/genes9120569

**Published:** 2018-11-23

**Authors:** Eduardo Zúñiga-León, Ulises Carrasco-Navarro, Francisco Fierro

**Affiliations:** Departamento de Biotecnología, Universidad Autónoma Metropolitana-Unidad Iztapalapa, Ciudad de Mexico 09340, Mexico; pgen10@hotmail.com (E.Z.-L.); ulises.c.n@gmail.com (U.C.-N.)

**Keywords:** Gene Ontology, KEGG pathways, enrichment analysis, proteomic analysis, plot visualization

## Abstract

The increasing number of OMICs studies demands bioinformatic tools that aid in the analysis of large sets of genes or proteins to understand their roles in the cell and establish functional networks and pathways. In the last decade, over-representation or enrichment tools have played a successful role in the functional analysis of large gene/protein lists, which is evidenced by thousands of publications citing these tools. However, in most cases the results of these analyses are long lists of biological terms associated to proteins that are difficult to digest and interpret. Here we present NeVOmics, Network-based Visualization for Omics, a functional enrichment analysis tool that identifies statistically over-represented biological terms within a given gene/protein set. This tool provides a hypergeometric distribution test to calculate significantly enriched biological terms, and facilitates analysis on cluster distribution and relationship of proteins to processes and pathways. NeVOmics is adapted to use updated information from the two main annotation databases: Gene Ontology and Kyoto Encyclopedia of Genes and Genomes (KEGG). NeVOmics compares favorably to other Gene Ontology and enrichment tools regarding coverage in the identification of biological terms. NeVOmics can also build different network-based graphical representations from the enrichment results, which makes it an integrative tool that greatly facilitates interpretation of results obtained by OMICs approaches. NeVOmics is freely accessible at https://github.com/bioinfproject/bioinfo/.

## 1. Introduction

Omics technologies are revolutionizing biological research by enabling genome-scale analysis of complex biological systems and processes [1]. Functional annotation of data from these approaches is essential to reduce the huge complexity of lists with hundreds to thousands of genes/proteins to a few processes or pathways in which they are involved, which will have more explanatory power than a simple list of identifiers. Several bioinformatic tools have been developed to perform functional annotations [2,3]. Over-representation analysis (ORA) is the most popular bioinformatic methodology to obtain significant functional information (enrichment) from sets of related genes/proteins [4]. The ORA method consists of searching in biological databases (e.g., Gene Ontology, GO [5] or Kyoto Encyclopedia of Genes and Genomes, KEGG [6]), using statistical testing to find biological terms, and functional annotations that are significantly enriched in a list of genes/proteins. The aim of the enrichment analysis is finding biological annotations that are over-represented in the query gene/protein list compared to what would be expected in a reference list (usually the whole proteome) [7]. In other words, if in a set of proteins certain biological processes or pathways are significantly enriched, the proteins with such signatures are likely to play these roles in vivo. However, in most cases, the results of these analyses are very long lists of biological terms or pathways associated to genes/proteins that are difficult to digest and interpret. Most available enrichment tools do not include comprehensible graphical visualizations and present the results as simple bar or pie chart plots, which do not allow insight into the functional relationships existing between the identified genes/proteins and the enriched GO terms and pathways [8]. As an example, a protein could be involved in three or more relevant biological processes or pathways, and a bar or pie chart plot cannot provide such information.

Network analysis has become an increasingly popular tool to deal with the complexity of large datasets of all sorts. The importance of using networks lies in their ability to reveal relationships between factors, rather than seeing them as isolated entities [9]. Intersection networks are bipartite networks which, when applied to biological systems, allow detection of multifunctional proteins, i.e., genes/proteins with more than one function and involved in more than one process or pathway.

Here we present NeVOmics (Network-based Visualization for Omics), a bioinformatic tool that facilitates the functional characterization of data from OMICs technologies such as transcriptomics and proteomics. NeVOmics has been developed in programming language Python, it integrates ORA methodology and network-based visualization with R packages, allowing the generation of four different types of graphical visualization to show the enrichment results. NeVOmics applies a hypergeometric statistical test to identify significantly enriched GO terms and pathways in a list of genes or proteins. The tool supports all organisms deposited in UniProt Knowledgebase (UniProtKB) and KEGG databases, and incorporates a functionality to assign pathways to organisms with no annotated genome information available from orthologous gene pathways deposited in the KEGG database.

## 2. Materials and Methods

### 2.1. General Features

NeVOmics has been developed in programming language Python. It integrates ORA methodology to obtain significantly over-represented GO terms and pathways in a list of genes/proteins. In addition, it uses R packages to provide network-based visualization to show the enrichment results. The code is configured to use updated information from UniProt-Gene Ontology Annotation (UniProt-GOA) [10,11], UniProtKB [12], and KEGG [6] databases. NeVOmics can be used in both Linux and Windows operating systems and it provides 13 additional protein lists from diverse organisms for the user to have the optionality to test the tool.

### 2.2. Usage

**Figure 1:**
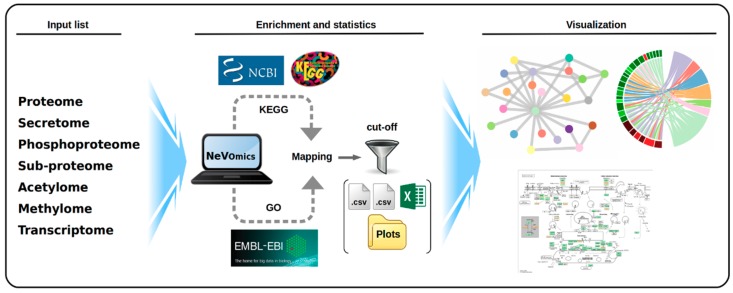
Schematic overview of the steps performed by NeVOmics (Network-based Visualization for Omics). The tool is composed of three main sections: input list, enrichment/statistics, and visualization. A connection is made to the UniProt-Gene Ontology Annotation (UniProt-GOA) and Kyoto Encyclopedia of Genes and Genomes (KEGG) databases, and then the content of the input list is analyzed. The enrichment/statistics section organizes and analyzes the data from the input list, retrieves information for all genes or proteins in the list, and the results are stored in files in .xlsx format. The National Center for Biotechnology Information (NCBI) logo indicates the source from which the reference proteomes are obtained by the KEGG database. The visualization section provides four types of graphical representation in .png format and high definition.

The flowchart for data processing is depicted in Figure 1. NeVOmics documentation is available for download from GitHub (https://github.com/bioinfproject/bioinfo/). It includes NeVOmics Python script, instructions for use, system requirements, and some examples.

NeVOmics can perform three different enrichment analyses using updated databases (Figure S1). The first analysis (1) is Gene Ontology using all information stored in UniProt-GOA (Complete GO Annotation) and UniProtKB Annotations. The second analysis (2) uses all annotations stored in the KEGG database to find relevant pathways. Finally, the third analysis (3) is more flexible because it can identify KEGG pathways from protein sequences by performing identity searches, and thus independent of the availability or not of annotated genome information for a particular organism. After executing each of the previous analyses, the information is stored in specific directories.

### 2.3. Input File

NeVOmics uses an input file in plain text containing a list of genes (KEGG gene ID) or proteins (UniProt Entry ID) obtained by any OMICs approach, such as comparative transcriptomics, proteomics, or proteome-specific methodologies (phosphoproteomics, acetylomics, etc.). The file can contain up to three columns (in Tabular format) depending on the results obtained in the “OMICs” experiment. The first column corresponds to the list of genes or proteins to be analyzed, for example a set of proteins showing abundance changes in a particular condition with respect to the control. The second column contains numerical values of expression/abundance or any other quantifiable value related to the study. The third column corresponds to a background list (e.g., all genes or proteins identified in the study) used as reference for statistics in the enrichment analysis; if the third column is absent in the input file the program automatically uses the entire proteome (UniProt-GOA) or KEGG pathways of the corresponding organism. In order to avoid ambiguities or inconsistencies in identifiers, NeVOmics allows only identifiers compatible with UniProtKB.

### 2.4. Annotations Sources

The Gene Ontology Annotation (GOA) database provides high quality electronic (mapping and automatic transfer of annotation to orthologous gene products) and manual (based on the literature) annotations to the UniProtKB (Swiss-Prot, TrEMBL, and PIR-PSD) using the standardized vocabulary of Gene Ontology. The GOA database contains information of nearly 60,000 species and more than 160,000 taxa, with more than 32 million annotations. NeVOmics uses the Gene Ontology of the Gene Ontology Consortium (GOC) [5], which is downloaded from: http://purl.obolibrary.org/obo/go.obo. It also uses the GOA association files, which are downloaded from File Transfer Protocol (FTP) in tab-delimited format (ftp://ftp.ebi.ac.uk/pub/databases/GO/goa/), and GO annotation in UniProtKB, which is provided as flat files that are downloaded from: http://www.ebi.ac.uk/uniprot/. In Swiss-Prot entries, only the manually annotated information is displayed. To view the complete GO annotation for a Swiss-Prot entry, the master copy of the data in the GOA association files is downloaded. GOA updates its annotation information weekly, while UniProtKB does it every four weeks. KEGG database covers information at different molecular levels. The KEGG Pathways database (https://www.genome.jp/kegg/pathway.html) is a collection of manually curated pathways, including information on molecular interactions, reactions, and network relationships. The KEGG database contains more than 24 million annotated genes and 530 pathways with more than six million pathway-linked genes.

### 2.5. Background

A background list of genes or proteins is essential for performing an adequate enrichment analysis and must be carefully chosen; a list with all the genes/proteins detected in any condition of the OMICs experiment is usually a valid background list. If the column of the background list (third column in the input file) is absent the program automatically uses the entire proteome in the case of UniProt-GOA or the entire KEGG database. For Gene Ontology analysis, NeVOmics builds background lists organized by category (Biological Process, Molecular Function, Cellular Component) of a specific organism for mapping the query protein list. NeVOmics downloads a GOA association file (ftp://ftp.ebi.ac.uk/pub/databases/GO/goa/proteomes/) from which it extracts all GO terms. For KEGG Pathways analysis NeVOmics builds a background file with all genes of a specific organism for mapping the query gene list.

### 2.6. Enrichment Analysis

NeVOmics analyzes the input list against the user’s preferred background and retrieves over-represented terms in the three GO categories (molecular function, biological process, cellular component) or in the KEGG pathways, with the genes/proteins classified by term or pathway. The tool adopts GeneMerge statistical algorithm to obtain the over-represented functions or categories in the input list [13]. A hypergeometric distribution test is performed to calculate the discrete probability of *x* (term or pathway) in a sample (gene/protein input list) of size *k* drawn from a background of size *N* (entire proteome by default or a background list provided by the user in the third column of the input file), where *m* corresponds to the total number of genes/proteins associated with a term or pathway (Figure S2). An FDR or modified Bonferroni correction of *p*-value is applied to identify the statistically more represented function annotations.

### 2.7. Output Files

NeVOmics generates three output files, stored in specific directories, containing lists of particular functions or KEGG pathways over-represented with statistical significance (Figure 1): (I) An edges file with two columns as ‘Source’ and ‘Target’, which contain the values of individual nodes that are linked together. (II) A nodes file with information of nodes ID, expression values, *p*-value and additional information for building plots. (III) Finally, an .xlsx file with enrichment analysis results for plotting in other network-based tools like Cytoscape [14] and Gephi [15]. NeVOmics automatically provides four types of graphical representations in high definition to facilitate the analysis and interpretation of results, these are: circular and random network, chord diagram [16], and UpSet plot [17]. These graphics are built with R packages as tidyverse, tidygraph, ggraph, igraph, viridis, circlize, RcolorBrewer, cowplot, networkD3, gridBase, ComplexHeatmap, and UpSetR. The graphics are configured according to the amount and type of data that resulted over-represented in the enrichment analysis.

### 2.8. Comparison of NeVOmics with Other Functional Enrichment Bioinformatic Tools

Gene Ontology and annotation sources for the analyses with NeVOmics were indicated in Section 2.4. GO terms from UniProt-GOA correspond to the version released on 18 October 2018. GOrilla and WebGestalt use GOC (http://www.geneontology.org/) for GO terms and as annotation source (http://geneontology.org/page/go-consortium-contributors-list); the update of 20 October 2018 was used in our analyses. g:Profiler uses also GOC for GO terms, and the annotation sources are Ensembl v93 (http://jul2018.archive.ensembl.org/index.html) and Ensembl Genomes v40 (http://ensemblgenomes.org/).

It is recommended that NeVOmics users make reference to the date of the analysis when publicizing their results, so that other researchers have information on the version of the UniProt-GOA release used for the analysis.

## 3. Results

NeVOmics is an integral tool with two major features: it allows enrichment analysis from a given list with data from some ‘OMICs’ experiment, and it builds different graphical representations in network form from the enrichment results. To test the functionality of NeVOmics we performed two case studies, using datasets from two different experimental OMICs procedures recently published. The first dataset comes from a platelet proteome of patients with early-stage cancer [18], and the second comes from a transcriptome analysis of mutants in tail module subunits of Mediator in *Arabidopsis thaliana*, a model system for research in plant biology [19].

### 3.1. Case Study 1: Enrichment Analysis of Differentially Expressed Platelet Proteins in Early-Stage Cancer

**Figure 2:**
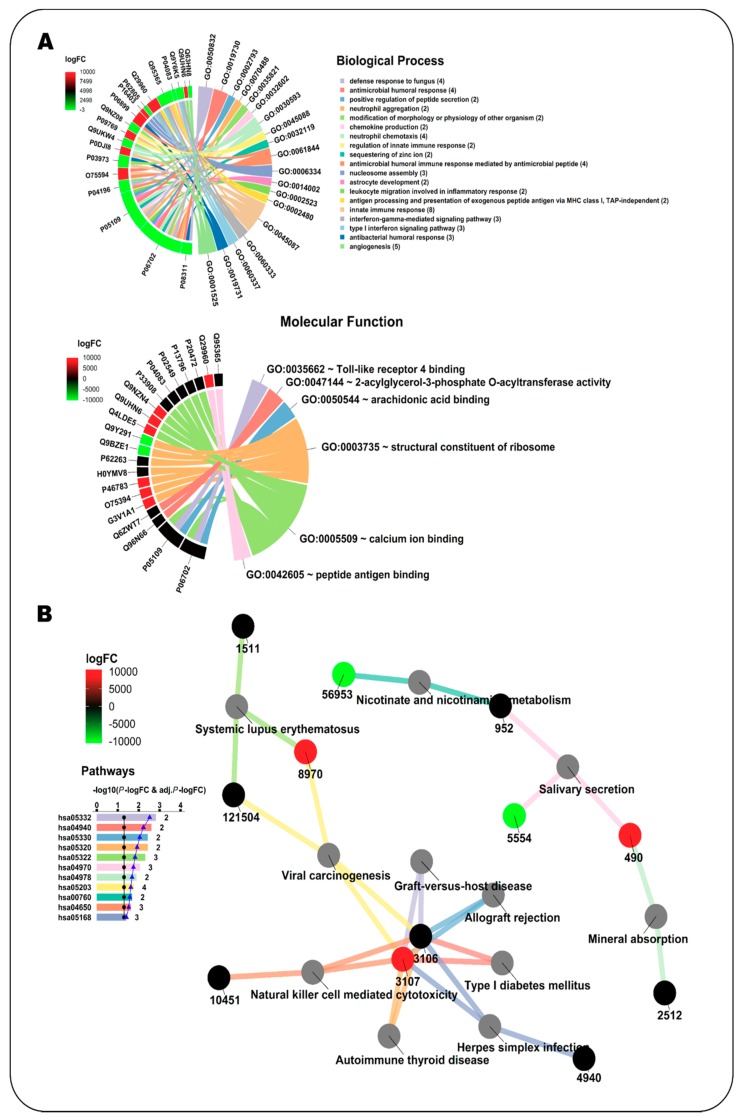
Enrichment analysis with NeVOmics of a dataset of platelet proteins showing significant abundance change (≥1.5-fold) in early-stage cancer vs. healthy condition (study conducted by Sabrkhany et al. [18]). (**A**) Chord diagram clustered by colors. Colors in the chords correspond to enriched biological processes (upper) or molecular functions (lower) (GO terms), linking each protein to the processes/functions to which it is related. Colors appearing in the sections of the outer circle beside each protein correspond to the abundance fold-change found in the study and are according to the heat map scale (logFC: log2 fold-change). (**B**) Network clustered by colors. Colors in the bar plot and the lines correspond to the identified enriched KEGG pathways, whereas colors in the protein identifier nodes correspond to the fold-change found in the study and are according to the heat map scale. The identifiers at the protein nodes are those provided by NCBI-GeneID. The bar plot indicates the −log10 of the *p*-value of each GO term, and the numbers at the end of the bars correspond to the total number of proteins detected for each term. The black line in the bar plot corresponds to the −log10 of 0.05, used as reference for *p*-value. The blue line on the bar plot corresponds to the −log10 of the adjusted *p*-value.

Platelets play an important role in tumor angiogenesis, growth and metastasis [20]. A study from Sabrkhany et al. [18] identified 4384 unique proteins expressed in platelets, of which 85 showed a significant abundance change (criteria Fc > 1.5 and *p* < 0.05) in early-stage cancer as compared to the control. Samples from 12 cancer patients (eight with lung cancer and four with pancreatic cancer) and 11 controls were used in the study. We analyzed with NeVOmics an input list with 61/24 platelet proteins which were ≥1.5-fold more abundant/≥1.5-fold less abundant, respectively, in platelets from individuals with early-stage cancer as compared to platelets from healthy individuals. As background we used a list with the total 4384 platelet-expressed proteins identified in this study. We carried out an enrichment analysis for Gene Ontology (with an FDR of 0.02) and KEGG Pathways (FDR 0.1). Nineteen biological processes were identified as enriched (Figure 2A), these fall mainly within the areas of inflammatory response, immune response, and cancer, according to UniProtKB annotations. In this category, 19 proteins were identified, of which nine (P08311, P06702, P05109, P04196, O75594, P03973, P06899, Q29960, and Q95365) are involved in more than two processes: P06702 has been shown to be differentially expressed in various types of cancer such as breast, colon, liver, gastric and non-small cell lung cancer, and is crucial for promoting cancer growth by recruitment of myeloid-derived suppressor cells. P06702 and P05109 usually form a heterodimer and are involved in inflammatory response and cancer development and progression, which explains their simultaneous presence in several enriched processes. In addition, five proteins were identified which are directly related to the angiogenesis process (GO:0001525), relevant in the early stages of cancer. For its part, in the category of molecular function, six functions resulted enriched (Figure 2A). In this category, 21 proteins were identified, of which two (P06702 and P05109) are involved in more than two functions. In the analysis with the KEGG database, 11 pathways were identified as enriched, mainly related to the immune response (Figure 2B). Only 12 proteins from the input list share pathways: Q95365/3106 and Q29960/3107 are involved in seven of the enriched pathways, whereas the other 10 are involved in one or two pathways.

### 3.2. Case Study 2: Enrichment Analysis of Gene Datasets from a Transcriptomic Study of *Arabidopsis thaliana* Mutants in Mediator Subunits

The mediator complex is a central component of transcriptional regulation in *Eukaryotes*. Whitney and Clint [19] studied the role of four *Arabidopsis* Mediator tail module subunits (MED2, MED5a/b, MED16, and MED23) by analyzing the transcriptome of mutants in each of the subunit-encoding genes. We used NeVOmics to perform an enrichment analysis of GO terms and KEGG pathways in four gene datasets corresponding each to the up- and downregulated genes found in a tail subunit mutant: med2 (341 genes), med5ab (283), med16 (723), and med23 (35). The total number of expressed genes identified in the Whitney and Clint’s study [19] was 18,842; we used this gene list to obtain compatible identifiers with the UniProtKB from The Arabidopsis Information Resource (TAIR) database, mapping 17,626 genes successfully. This list of 17,626 gene products was used as background in the enrichment analysis.

**Figure 3:**
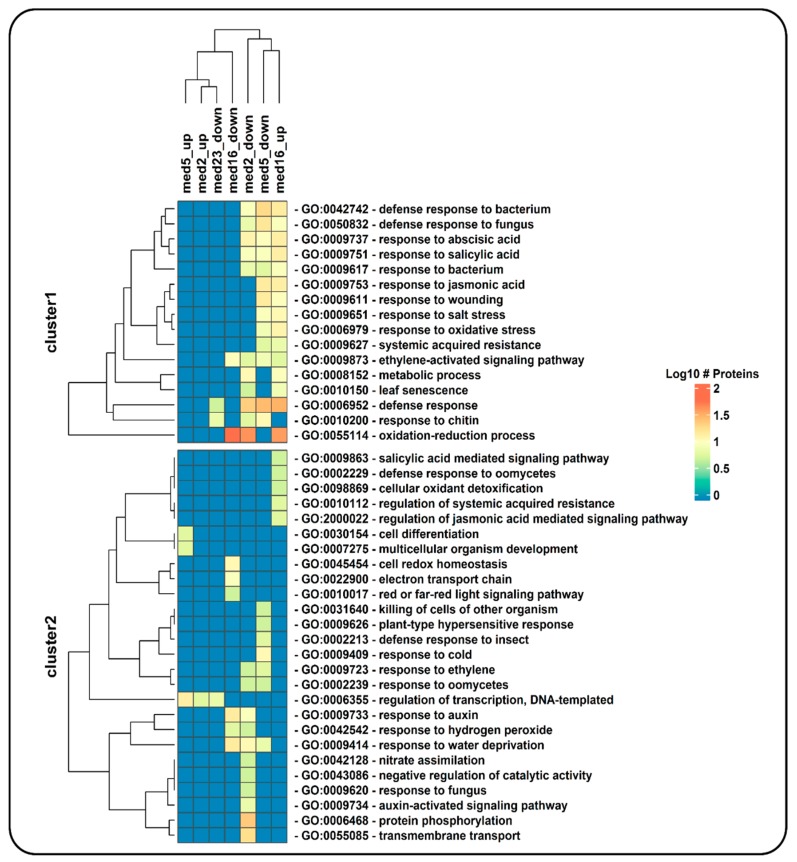
Hierarchical clustering of enriched GO terms in four gene datasets corresponding to up- and downregulated genes identified in a transcriptomic analysis of mutants of Mediator tail module subunits from *Arabidopsis* (study conducted by Whitney and Clint [19]). Only GO terms belonging to the category of ‘biological process’ are shown, and among them only those including more than five genes from the datasets. Each cell in the heatmap is colored according to the number of proteins (log10 of this number) detected by NeVOmics in the corresponding enriched GO term (rows) and in the specific condition (columns); “up” and “down” tags in column names correspond to “upregulated genes” and “downregulated genes”, respectively.

To compare our results with those obtained by Whitney and Clint [19], we used an FDR of 0.05 to consider a GO term enriched, the same they used when performing their enrichment test with DAVID v6.8 [21]. In total, 137 unique GO terms were enriched with NeVOmics in the four conditions, they are represented in a clustered heatmap that includes all up- or downregulated identified gene products (Figure S3 and Table S1). In Figure 3 is shown a clustered heatmap elaborated with 42 enriched GO terms that contained more than five proteins each (data also in Table S2). The raw data generated by NeVOmics are stored in several .xlsx format output files from which information can be extracted and used to make custom visualizations using other packages or programs. Whitney and Clint [19] identified only one enriched GO term (response to bacterium) in the condition of “downregulated genes in the med2 mutant”, and none in the “upregulated genes”, whereas with NeVOmics we were able to detect 54 GO terms in the “downregulated genes” condition and five in the “upregulated genes” one (Figure S3 and Table S1). Regarding the med5 mutant, Whitney and Clint [19] detected 16 GO terms in “downregulated genes”, and five in “upregulated genes”, whereas with NeVOmics we found 53 GO terms in “downregulated genes” and 12 GO terms in “upregulated genes”. In all cases, all GO terms identified by Whitney and Clint [19] with DAVID v6.8 were also detected with NeVOmics. In the new GO terms identified by NeVOmics there are more processes associated with the regulation of transcription along with different response mechanisms, for example: regulation of transcription by RNA polymerase II, DNA-templated regulation of transcription, DNA-templated response to oxidative stress, response to water deprivation, response to salt stress, and response to abscisic acid (Figure S3 and Table S1). In the med16 mutant, NeVOmics detected 22 enriched GO terms in “downregulated genes” and 53 in “upregulated genes”. In Figure 3, the cluster 1 contains several enriched GO terms detected mainly in the mutants med2, med5 (“downregulated genes”) and med16 (“upregulated genes”). The GO terms included in cluster 1 represent processes involved in response and defense to several factors. Finally, in the med23 mutant, whose transcriptome revealed only 35 downregulated genes, NeVOmics identified several GO terms that are absent in the Whitney and Clint work (Figure S3 and Table S1). In summary, most of the terms detected by NeVOmics are related mainly to some response and defense to several factors, or related to small molecules such as auxins, gibberellins, abscisic acid, ethylene, brassinosteroids, jasmonic acid, and salicylic acid (Figure S3 and Table S1), which play a major role in seed maturation and germination, as well as in adaptation to abiotic environmental stresses [19].

**Figure 4:**
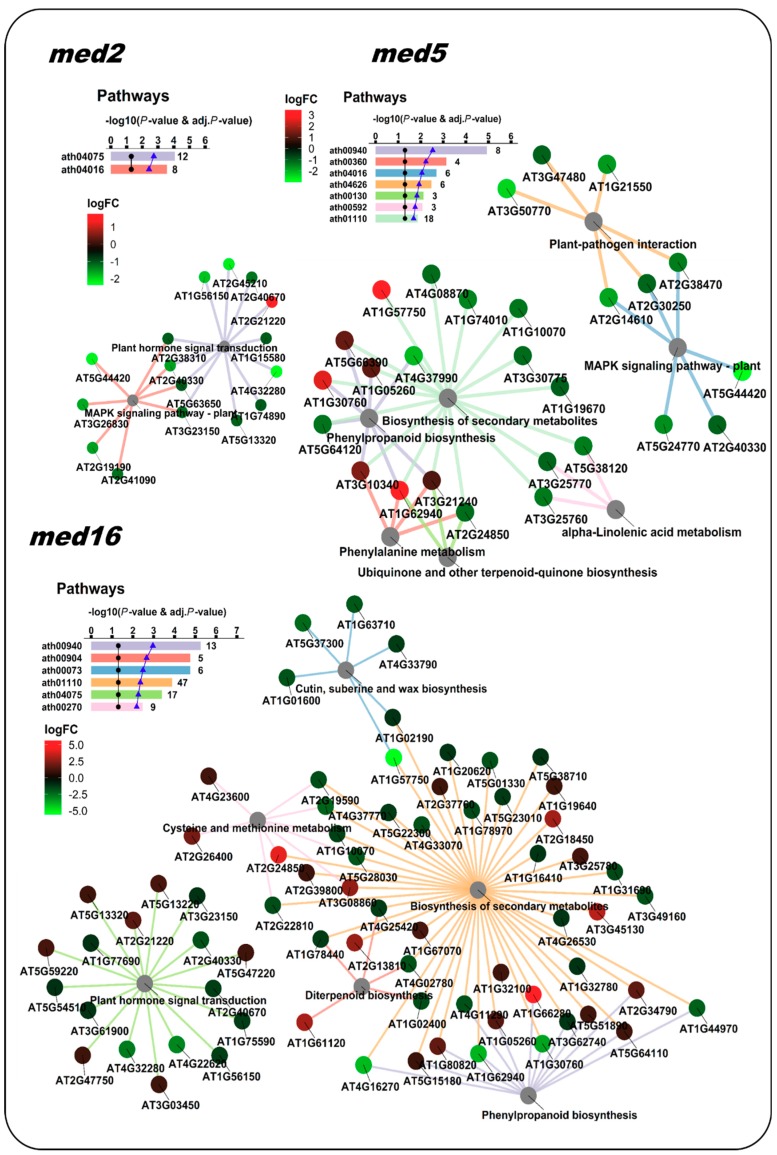
NeVOmics enrichment analysis of KEGG pathways using the same datasets as in Figure 3. med5 and med16 mutants shared several enriched pathways. These networks enable the effective representation of associated data, such as the number of elements in the aggregates and intersections, and the quick identification of proteins involved in more than one process or pathway. The ratio of each gene product abundance in the corresponding mutant over the control is indicated by the node color, according to the fold-change color scale (logFC: log2 fold-change). The bar plot indicates the −log10 of the *p*-value and the number of proteins found for each of the enriched pathways.

Regarding the Pathway analysis, Whitney and Clint [19] identified enriched pathways in both “upregulated” and “downregulated genes” conditions from all med mutants, except in the med23 mutant. They found a total of eight enriched KEGG pathways: ath04075 (Plant hormone signal transduction) and ath00945 (Stilbenoid, diarylheptanoid and gingerol biosynthesis) were found in the condition of “downregulated genes in the med2 mutant”; ath01110 (Biosynthesis of secondary metabolites), ath00073 (Cutin, suberine and max biosynthesis) and ath00904 (Diterpenoid biosynthesis) were found in “downregulated genes in the med16 mutant”; ath00940 (Phenylpropanoid biosynthesis) was found in “upregulated genes in the med5 mutant”; and two other pathways were found additionally, ath04626 (Plant-pathogen interaction) and ath00592 (alpha-Linolenic acid metabolism). In our analysis, NeVOmics identified all pathways described by Whitney and Clint [19] except for ath00945, and additionally it detected three more pathways: ath00270 (Cysteine and methionine metabolism), ath00130 (Ubiquinone and other terpenoid-quinone biosynthesis), and ath04016 (MAPK signaling pathway plant) (Figure 4).

### 3.3. Comparison and Advantages of NeVOmics over Other Function Enrichment Analysis Tools

NeVOmics is designed to perform enrichment analysis using updated information of the two main annotation databases (GO and KEGG) automatically, making it unnecessary to have to update the information by other means. NeVOmics has the versatility to use UniProt-GOA and UniProtKB separately as databases, to obtain complete (electronic + manually-curated) and only manually-curated information, respectively, in order to get as much information as possible. There exist other available tools that can perform enrichment analysis on gene or protein datasets, however, these tools update their information on Gene Ontology less frequently and thus are less reliable for performing function enrichment analysis [22]. Some of these tools do not include more than two databases, or do not offer graphical representations to aid in the visualization of results (Table S3).

NeVOmics is also very versatile regarding the organisms that can be submitted to analysis, it supports all organisms deposited in UniProtKB (https://www.uniprot.org/proteomes/) and KEGG (https://www.kegg.jp/kegg/catalog/org_list.html) databases, and actually any organism can be used, even those lacking annotated genome information, due to NeVOmics functionality to assign pathways searching orthologous gene pathways deposited in the KEGG database (see Materials and Methods, Section 2.2). In contrast, other tools such as g:Profiler [23], GOrilla [24], GOEAST [25], and WebGestalt [26] have the limitation of excluding non-model organisms (Table S3).

**Figure 5:**
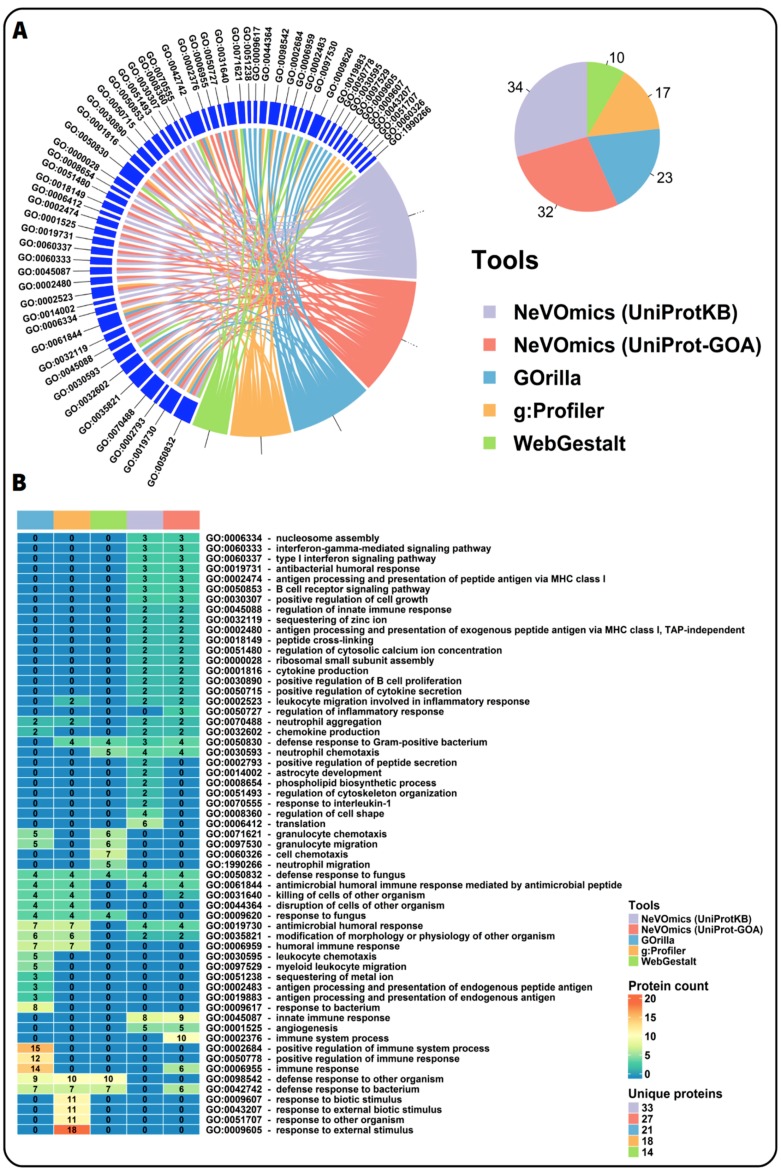
Comparison of NeVOmics with other enrichment tools. (**A**) Chord diagram showing a comparison of enriched GO terms in the category of biological process detected by NeVOmics and three other tools using the data of Case Study 1 previously described. GOEAST was also included in the analysis, however it did not recognize the identifiers and generated an error message. The pie chart shows the total number of GO terms identified by each tool. (**B**) Heatmap showing protein counts for each GO term (rows) obtained with the different tools (columns). Each cell is colored according to the number of proteins detected by a tool in the specific GO term. “Unique proteins” indicates the total number of unique proteins identified by each tool.

To test whether NeVOmics is able to offer different and/or more complete/accurate results in enrichment analysis when compared to other tools, we performed a comparison test between NeVOmics and some publicly available enrichment tools: g:Profiler (version beta), Gorilla (version not available), GOEAST (version 1.20), and WebGestalt (version 2019 beta). Unlike NeVOmics and WebGestalt, Gorilla, and g:Profiler present flexibility limitations in the adjustment of some parameters during the analyses, for instance they control the *p*-value threshold not allowing to set up other custom-chosen FDR values. Despite these differences, we tried to make the analyses as comparable as possible. In the comparison test we searched for enriched GO terms in the category of Biological Processes using the data of Case Study 1, with an FDR of 0.05 for NeVOmics. Although WebGestalt allows to control several parameters, when selecting an FDR of 0.05 it does not detect enriched GO terms, therefore, in order to include this tool in the analyses we used its “TOP” option for 10 GO enriched terms. As shown in Figure 5, NeVOmics attained greater coverage of identified GO terms in comparison to other tools. With NeVOmics some GO terms were detected which went undetected to other tools, and the opposite also occurred in some cases. The terms detected by NeVOmics and absent in the analyses with other the tools were: GO:0002523 (leukocyte migration involved in inflammatory response), GO:0050727 (regulation of inflammatory response), GO:0030890 (positive regulation of B cell proliferation), GO:0030307 (positive regulation of cell growth), GO:0001525 (angiogenesis), GO:0070555 (response to interleukin-1), and GO:0060333 (interferon-gamma-mediated signaling pathway), all of them related to immune response processes and cancer. On the other hand, NeVOmics, GOrilla, and WebGestalt did not detect GO terms related to response to environmental stimulus (GO:0009607, GO:0043207, and GO:0009605), which appeared highly enriched in g:Profiler. Therefore, there are differences between tools in the number and identity of detected GO terms and also in the amount of proteins that each program identifies. Three possible reasons that could determine these differences are: the source of annotation and GO definitions, the regularity with which these databases are updated, and the statistical approach to obtain enriched terms. All the other tools detected fewer proteins than NeVOmics: WebGestalt covered 41% of the total number of proteins detected by NeVOmics, g:Profiler covered 52% and GOrilla 61%, which indicates that our tool can provide more complete functional information. In addition, NeVOmics automatically generates a set of graphs to facilitate the visualization of results. NeVOmics can generate four different graphics per analysis depending on the type and amount of data, whereas some of the analyzed tools generate only simple visualizations.

## 4. Discussion

NeVOmics is an enrichment analysis tool executable in the command line, able to analyze from five to thousands of identifiers in gene/protein datasets. It can perform three different types of analyses (described in Section 2.2), using automatically updated information from the main annotation resources for Gene Ontology and pathways (GOA and KEGG). There is no requirement to download additional databases or any other resources.

NeVOmics uses ORA methodology and has been designed to facilitate the analysis and interpretation of large amounts of data, such as those obtained by high-throughput OMICs techniques, from a very wide range of organisms. The tool also allows the inclusion of expression/abundance data or any other experimentally-obtained quantifiable value, such as phosphorylation or other protein modifications, which provides information about how genes/proteins are up/downregulated in different biological conditions, thus allowing a better understanding of their role in the processes and pathways that are enriched.

As proof of concept, we performed different tests with datasets from distantly related organisms such as humans and *Arabidopsis thaliana*, with similarly successful results. In addition, we compared our tool to other available enrichment tools by analyzing a protein dataset from platelets of early-stage cancer patients. NeVOmics proved to be more sensitive and was able to identify a higher number of cancer-related processes (GO terms) and proteins participating in these processes than any of the other tools. Nevertheless, we encourage researchers to use more than one tool when performing Gene Ontology and pathway enrichment analysis, in order to make comparisons and verification of results and thus be able to make more accurate conclusions.

In addition to its capabilities for functional annotation by enrichment analysis, NeVOmics builds and provides different types of network-based graphical visualizations to get a better comprehension of experimental results from OMICs technologies, and to illustrate and communicate the information in an integral form. In future versions, we aim to include the Reactome (https://reactome.org/) and NCG (http://ncg.kcl.ac.uk/) databases (only for *Homo sapiens*). We will also work to include new features such as enrichment of protein domains and building of networks based on protein-protein interactions.

## Supplementary Materials

The following materials are available online at: http://www.mdpi.com/2073-4425/9/12/569/s1 and https://github.com/bioinfproject/bioinfo/. Figure S1: Description on how to run NeVOmics on the command line in Windows and Linux. Figure S2: Hypergeometric distribution. Figure S3: Heat map of all GO terms detected by NeVOmics generated with Table S1. Table S1: Data matrix in .csv format with 137 unique GO terms enriched by NeVOmics in Case Study 2. Table S2: Data matrix in .csv format with GO terms enriched by NeVOmics in Case Study 2, with more than five proteins each. Table S3: Comparison of NeVOmics features with other functional enrichment tools. The supplementary material also includes thirteen files (File_S1–File_S13) in .tsv format with protein lists from *Homo sapiens* and *Arabidopsis thaliana* used in Case Studies 1 and 2, respectively, to test NeVOmics.
