# Supplementary material for: NeVOmics: An Enrichment Tool for Gene Ontology and Functional Network Analysis and Visualization of Data from OMICs Technologies
Source: Genes (Basel). 2018 Nov 23;9(12):569. doi: 10.3390/genes9120569 (PMC6316660; doi:10.3390/genes9120569)
Supplement: Supplementary file 1 [file genes-09-00569-s001.zip › Supplementary_Material/Supplementary_Material.pdf]

Supplementary Material

Supplementary Figure S1: NeVOmics can perform three different enrichment analysis using updated databases. The first analysis is Gene Ontology using all information stored in UniProt-GOA and UniProtKB. The second analysis uses all annotations stored in the KEGG database to find relevant pathways. The third analysis identify KEGG Pathways in not annotated organism from protein sequences.

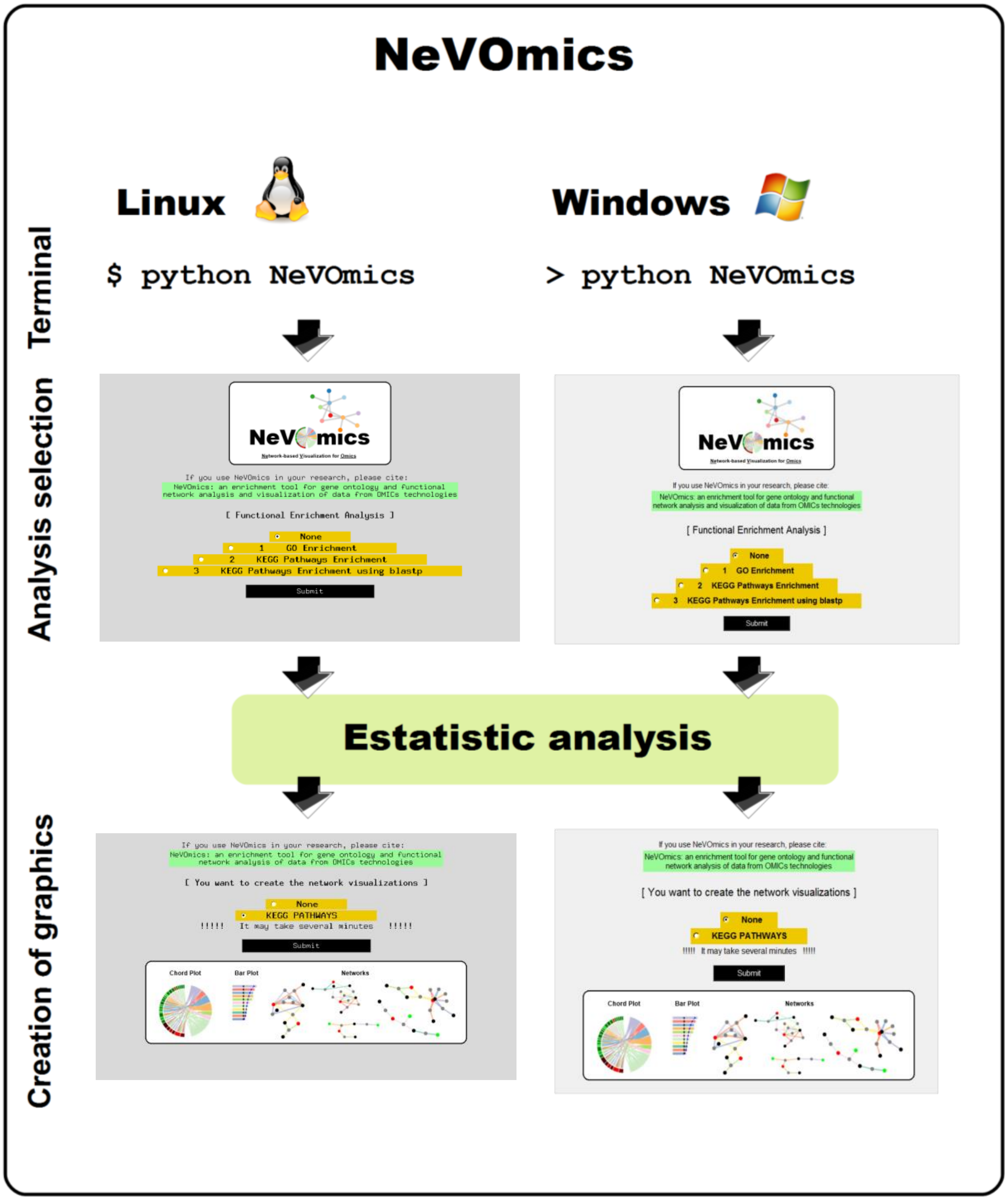

Supplementary Figure S2: Hypergeometric Distribution.

$$p - value = \frac{\binom{m}{x}\binom{N-m}{k-x}}{\binom{N}{k}}$$

Variable	Description
**N**	The total number of genes/proteins with some annotation (background) in the Gene Ontology.
m	The total number of genes/proteins from background annotated for some GO term/Pathway (e.g. Biological Process: signaling, GO:0023052).
K	Size of the selection. Number of genes/protein associated to at least one "Biological Process" in the Gene Ontology.
x	The number of genes/proteins inside the selection, associated with the process "signaling".

Supplementary Figure S3: 137 unique GO terms detected with NeVOmics in the four conditions of Case study 2. These data are represented in a clustered heatmap that includes all up- or down-regulated identified gene products.

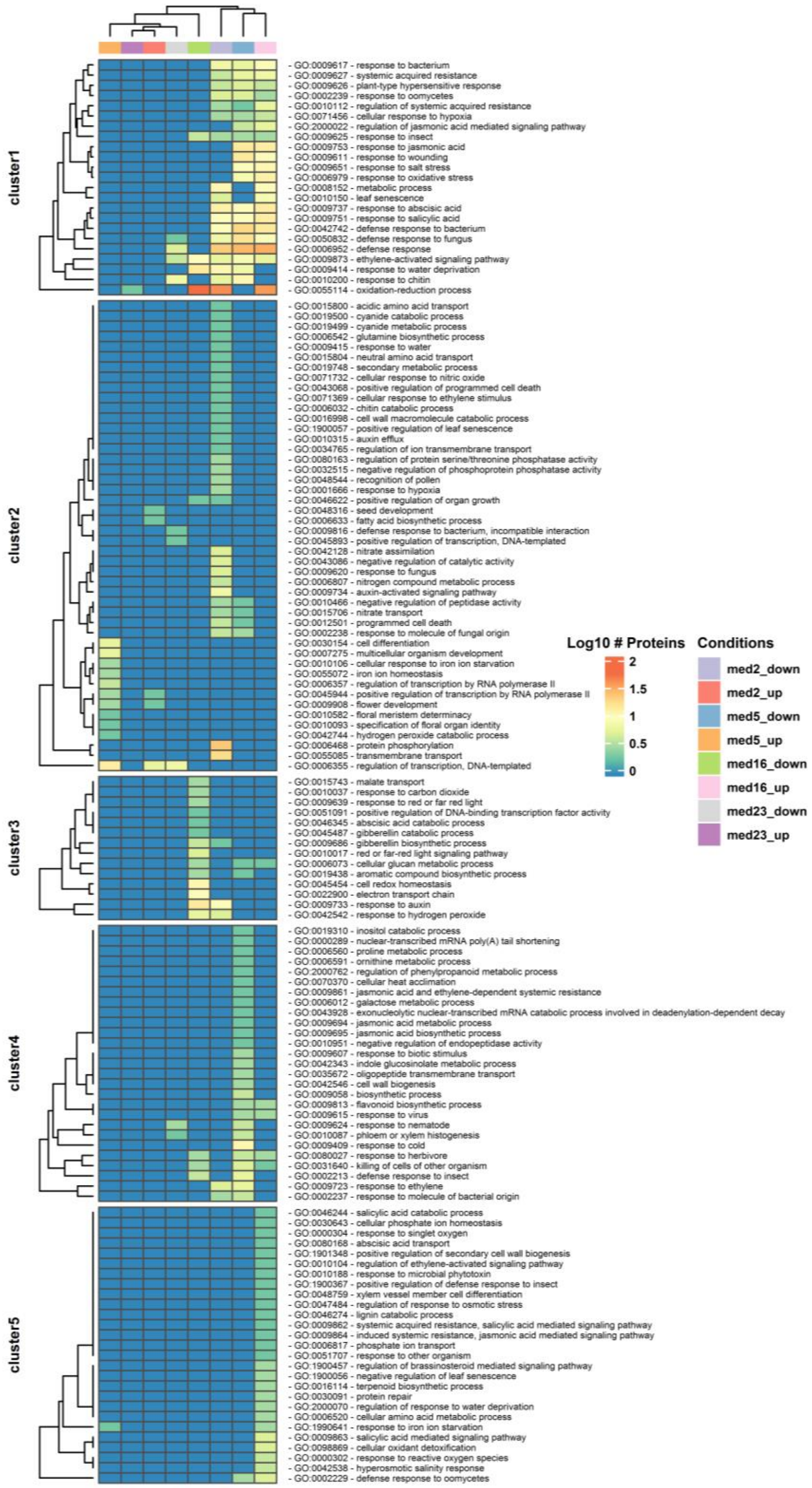

Supplementary Table S1. Data matrix in .csv format with 137 unique GO terms enriched by NeVOmics in Case study 2.

Supplementary Table S2. Data matrix .csv format with GO terms enriched by NeVOmics in Case study 2, with more than five proteins each.

Supplementary Table S3. Comparison of NeVOmics features with other functional enrichment tools.

Enrichment tool	Organism	GO Ontology	KEGG Pathways	Statistical method	Correction	Graphical Visualization	Time	Ref
**g:Profiler***	213 (species)	A	L	H	FDR	Network	1 min	[1]
**GORILLA***	8	A	Na	mHG	FDR	DAG	30 sec	[2]
**DAVID***	Unknown	A	A	F	FDR Bonferroni	NA	2 min	[3]
**GOEAST***	60 (species)	A	Na	F, H, Chi-square	FDR Bonferroni	DAG	20 min	[4]
**WebGIVI***	Unknown	A	Na	F	Unknown	Network and Graphics	3 min	[5]
**WebGestalt***	12	A	A	H	FDR	DAG Network	3 min	[6]
**BiNGO**	Unknown	A	Na	H	FDR Bonferroni	Network	Unknown	[7]
**Funrich**	Annoted species	A	Na	Unknown	Unknown	Network and Graphics	Unknown	[8]
**NeVOmics**	**Annoted species**	**A**	**A**	**H**	**FDR Bonferroni**	**Network and Graphics**	**Variable**	

A: Applicable
Na: Not Applicable
L: Limited, not applicable to all organisms
H: Hypergeometric distribution
F: Fisher's exact test
FDR: False Discovery Rate
DAG: Directed Acyclic Graph
mHG: minimum Hypergeometric
***** : Web tools

References

1. Reimand J.; Arak, T.; Adler, P.; Kolberg, L.; Reisberg, S.; Peterson, H.; Vilo, J. g:Profiler-a web server for functional interpretation of gene lists (2016 update). *Nucleic Acids Res. 2016,* 44, W83-9. [PubMed] [CrossRef]
2. Eden, E.; Navon, R.; Steinfeld, I.; Lipson, D.; Yakhini, Z. GOrilla: a tool for discovery and visualization of enriched GO terms in ranked gene lists. *BMC Bioinformatics.* 2009, 10, 48. [PubMed] [CrossRef]
3. Huang D.W.; Sherman, B.T.; Tan Q.; Collins, J.R.; Alvord, W.G.; Roayaei, J.; Stephens, R,; Baseler, M.W.; Lane, H.C.; Lempicki, R.A. The DAVID Gene Functional Classification Tool: a novel biological module-centric algorithm to functionally analyze large gene lists. *Genome Biol.* 2007, 8, R183. [PubMed] [CrossRef]
4. Zheng, Q.; Wang, X.J. GOEAST: a web-based software toolkit for Gene Ontology enrichment analysis. *Nucleic Acids Res.* 2008, 36, W358-63. [PubMed] [CrossRef]

5. Sun, L.; Zhu, Y.; Mahmood, A.S.M.; Tudor, C.O.; Ren, J.; Vijay-Shanker, K.; Chen, J.; Schmidt, C.J. WebGIVI: a web-based gene enrichment analysis and visualization tool. *BMC Bioinformatics.* 2017, 18, 237. [PubMed] [CrossRef]
6. Zhang, B.; Kirov, S.; Snoddy, J. WebGestalt: an integrated system for exploring gene sets in various biological contexts. *Nucleic Acids Res.* 2005, 33, W741-8. [PubMed] [CrossRef]
7. Maere, S.; Heymans, K.; Kuiper, M. BiNGO: a Cytoscape plugin to assess overrepresentation of Gene Ontology categories in Biological Networks. *Bioinforma. Appl.* 2005, 21, 3448–3449. [PudMed] [CrossRef]
8. Mohashin, P.; Shivakumar, K.; David, C.; Riccardo, A.; Ching-Seng, A.; Philip, A.; Arsen O.B.; Alberto, B.M.; Giovanni, C.; Aled, C.; Federica, C.; Dolores, D.V.; Juan, M.F.P.; Pedro, F.; Pamali, F.; Simona, F.; Yong, S.G.; An, H.; Esther, N-'t.H.; Nunzio, I.; Kenneth, K.; Thomas, K.; Joanna, K.; Igor, V.K.; Tommaso, L.; Yaxuan, L.; Alicia, Ll.; Taral, R.L.; Sayantan, M.; Francesca, M.; Anders, Ø.; Theocharis, P.; Tushar, P.; Héctor, P.; Stefano Pluchino, Simona, P.; Goran, R.; Felix, R.; Susmita, S.; Cristiana, S.; Allan, S.; Clotilde, T.; Martijn, J.C. van H.; Marca, W.; Joanne, L.W.; Kening, Z.; Suresh, M.. A novel community driven software for functional enrichment analysis of extracellular vesicles data. *J. Extracell. Vesicles.* 2017, 6, 1321455. [PubMed] [CrossRef]

Files in .tsv format with protein lists from *Homo sapiens* and *Arabidopsis thaliana* used in Case study 1 and 2, respectively, to test NeVOmics.

Supplementary File_S1: Early stage cancer list used in Case study 1

Supplementary File_S2: med2 condition, Up/Down regulated genes used in Case study 2

Supplementary File_S3: med2 condition, Down regulated genes used in Case study 2

Supplementary File_S4: med2 condition, Up regulated genes used in Case study 2

Supplementary File_S5: med5 condition, Up/Down regulated genes used in Case study 2

Supplementary File_S6: med5 condition, Down regulated genes used in Case study 2

Supplementary File_S7: med5 condition, Up regulated genes used in Case study 2

Supplementary File_S8: med16 condition, Up/Down regulated genes used in Case study 2

Supplementary File_S9: med16 condition, Down regulated genes used in Case study 2

Supplementary File_S10: med16 condition, Up regulated genes used in Case study 2

Supplementary File_S11: med23 condition, Up/Down regulated genes used in Case study 2

Supplementary File_S12: med23 condition, Down regulated genes used in Case study 2

Supplementary File_S13: med23 condition, Up regulated genes used in Case study 2
